# Corticopuncture Facilitated Microimplant-Assisted Rapid Palatal Expansion

**DOI:** 10.1155/2018/1392895

**Published:** 2018-12-06

**Authors:** Selly Sayuri Suzuki, Laila Fernanda Souza Braga, Denise Nami Fujii, Won Moon, Hideo Suzuki

**Affiliations:** ^1^Department of Post-Graduation in Orthodontics, São Leopoldo Mandic Institute and Research Center, Campinas, SP, Brazil; ^2^School of Dentistry, Section of Orthodontics, University of California, Los Angeles, CA, USA

## Abstract

**Introduction:**

Microimplant-assisted rapid palatal expansion (MARPE) has been considered an alternative to avoid extensive surgical procedures. In order to obtain skeletal results of MARPE, force should be enough to overcome areas of resistance and the first one that is required to be disrupted is the midpalatal suture, which becomes increasingly interdigitated after adolescence.

**Objective:**

The present study aimed at providing a novel approach using a minimally invasive method called corticopuncture (CP) in association with MARPE illustrated by a case report of a 35-year-old Brazilian female Caucasian patient presenting maxillary transverse deficiency.

**Method:**

Treatment plan started with an orthopedic correction of the transverse problem using a MARPE device. After many unsuccessful attempts to activate MARPE, corticopunctures were performed along the midpalatal suture. CP procedure at the midpalatal suture included 8 perforations (2 mm apart), performed after previous predrilling followed by miniscrew insertion (5 mm thread length and 1.8 mm diameter).

**Results:**

After CP and new activation protocol, the opening of the midpalatal suture was observed by CBCT images, showing skeletal results, suture split of 3.14 mm (premolar area) and 2.06 (molar area), an increase of 4.3 mm (premolar) and 3.03 mm (molar) in basal bone width, 4.43 mm (premolar) and 3.1 mm (molar) in cortical bone width, and minimal dental effects (mean of 1.2° of tooth tipping).

**Conclusion:**

The combination of MARPE and corticopuncture method was proved to be a nonsurgical treatment option to correct maxillary transverse deficiency in an adult patient. CP was able to weaken suture interdigitation thus facilitating the split.

## 1. Introduction

According to MacGinnis et al. [1], prevalence of maxillary transverse deficiency has been found to be between 8% and 23% in mixed and deciduous dentitions and less than 10% in adults. This malocclusion represents a common problem found in clinical orthodontics. Another study cited that 9.4% of the entire population and approximately 30% of adult orthodontic patients present maxillary transversal deficiency related to posterior crossbite [2].

Maxillary orthopedic expansion in adult patients through conventional devices has been considered rarely successful. The cause is commonly related to the fusion (or progressive calcification) of the midpalatal suture and the increased interdigitation of craniofacial sutures, making it more resistant to split as age progresses [3, 4].

Expansion forces transmitted to teeth in traditional rapid palatal expansion (RPE) devices can create undesirable dental effects rather than real bone expansion, especially in adult patients with more rigid interdigitations of the midpalatal suture [5]. Thus, dental inclination and bending of the alveolar bone are unavoidable effects in these cases. In addition, limitations and side effects of conventional RPE are common, such as failure in expansion or limited skeletal expansion, pain, tissue swelling, buccal inclination of the posterior teeth, gingival retraction, root resorption, and relapse [6]. Buccal inclination of the posterior teeth commonly leads to a clockwise rotation of the mandible and consequently bite opening which are unfavorable in dolichofacial patients [1].

Surgically assisted rapid palatal expansion is the treatment choice to overcome limitations of conventional RPE devices by osteotomy procedures in which the maxillary basal bone is separated from its main structures of the skull, allowing rapid expansion with mainly skeletal effects in adult patients [7–9]. In recent years, micro-implante assisted rapid palatal expansion (MARPE) has been developed to avoid unwanted dental effects and achieve pure skeletal results, especially indicated for patients at the end of growth or adults who are reluctant to the surgical procedure [10].

Although MARPE technique potentially represents a nonsurgical alternative to treatment of maxillary atresia in patients in the final stage of growth and adults, it may present limitations, due to the increase in interdigitation of the midpalatal suture that occurs after puberty. With advancing age, sutures are usually heavily interdigitated through the ossification process [3]. Recently, many studies have proposed methods to stage skeletal maturation of the midpalatal suture, since it is advocated that the amount of the skeletal or dentoalveolar effect of the maxillary expansion procedure may be correlated to the maturation of the midpalatal suture [11–14]. In order to obtain skeletal results of MARPE, force should be enough to overcome areas of resistance located in the midface such as piriform aperture pillars, zygomatic buttresses, pterygoid junctions, and the midpalatal suture, the first one that requires to be disrupted [15, 16]. Therefore, it would be advantageous to reduce any possible area of bone resistance during maxillary expansion with MARPE [17].

Currently, regional acceleratory phenomenon (RAP) induced by surgical trauma has received great attention by acting directly on bone remodeling, accelerating tooth movement, and consequently reducing orthodontic treatment time [18]. RAP occurs when a mechanical bone trauma is performed by surgical intervention and is generally limited to the alveolar bone. This process initiates and potentiates a normal bone repair process, increasing the turnover of the alveolar bone, and finally promotes faster tooth movement [19]. May researches have been conducted in order to find minimally invasive procedures for RAP induction. Among the surgical methods that have been studied are corticotomy [20], bone microperforations [21, 22], piezocision [23], corticopuncture [24], and corticision [25].

In the literature, a few case reports demonstrated the use of the corticotomy method as an aid in the expansion of the upper arch, called CAE (corticotomy-assisted expansion) in treating maxillary transverse deficiency [26, 27]. The recommended corticotomy procedure is bilateral decortication of the alveolar, buccal, and palatine bones and the use of dental expanders. According to Hassan et al., the corticotomy method during expansion can reduce the resistance to expansion, lead to faster tooth movement, and lessen the side effects of conventional expansion [28].

The aim of this study was to present a case of an adult patient treated with a method called corticopuncture facilitated microimplant-assisted rapid palatal expansion (CP + MARPE) which stands for an association of microimplant-assisted rapid palatal expansion and perforations called corticopunctures performed along the midpalatal suture, in order to reduce the resistance and optimize its opening.

## 2. Case Report

### 2.1. Diagnostics

A 35-year-old female patient presenting a unilateral skeletal crossbite in maximum intercuspation attended the postgraduation orthodontic clinics with the main concern of “crooked smile” and had never undergone orthodontic treatment.

The patient was diagnosed with mesofacial facial type, presenting facial asymmetry in the lower facial third, with mandibular deviation to the right side in relation to the facial midline. During smile, an increased buccal corridor was observed, confirming maxillary constriction and crossbite on the right side. Profile analysis showed a straight orthognathic profile.

**Figure 1:**
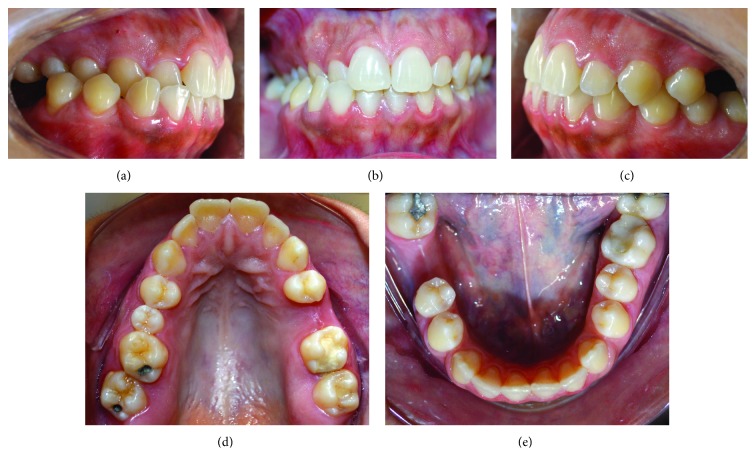
Initial intraoral images: (a) right side, (b) frontal, (c) left side, (d) occlusal view of upper arch, and (e) occlusal view of lower arch.

**Figure 2:**
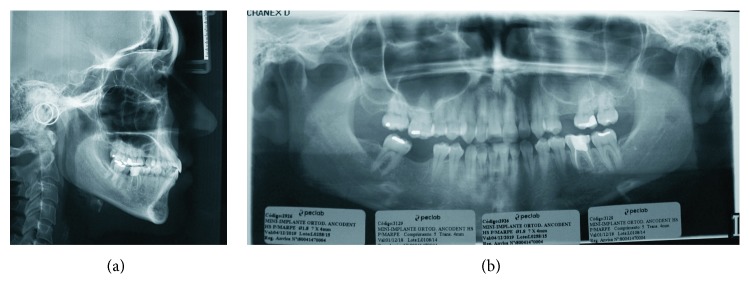
Lateral X-ray (a). Panoramic X-ray (b).

Intraoral photos showed canines in Class I relationship with unilateral crossbite on the right side, 1 mm overjet, 2 mm overbite, triangular shape of the upper arch, and oval shape in the lower arch (Figure 1). Upper right second premolar showed a shape anomaly, and teeth 25 and 46 were absent. In clinical examination, a functional shift was observed when evaluating mandibular movement patterns so that centric relation and centric occlusion were not coincident. Lateral and panoramic x-rays show good inclination of upper incisors, lower incisors slightly proclined, and absence of all third molars (Figure 2).

**Figure 3:**
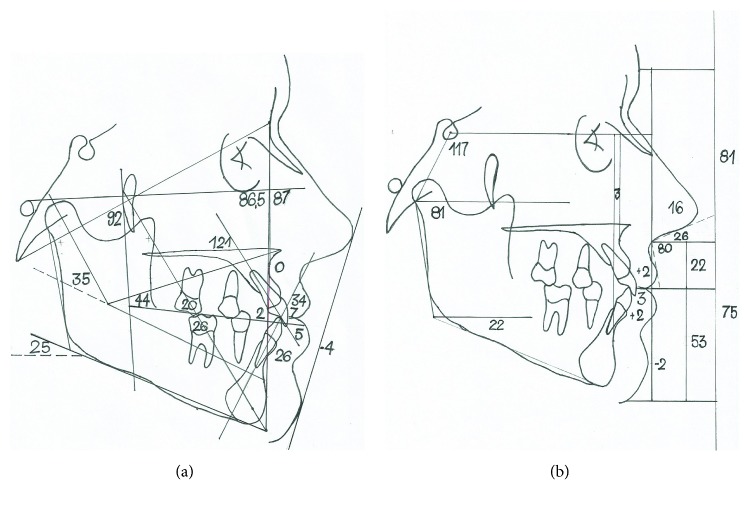
Ricketts cephalometric and soft and hard tissue integration analyses.

Ricketts cephalometric analysis indicated a mesofacial, skeletal class I patient with protruded and slightly extruded lower incisors, slightly protruded upper incisors, and mesialized mandibular first molars. In the soft and hard tissue integration analysis, centroid-based wits showed skeletal Class I and all angles within the normal limits, determining a neutral growth. The middle and lower facial thirds were balanced, and the nose, lower lip, upper lip, and soft chin are well positioned. Lip sealing was presented (Figure 3).

### 2.2. Treatment Plan and Progress

#### 2.2.1. MARPE Appliance

**Figure 4:**
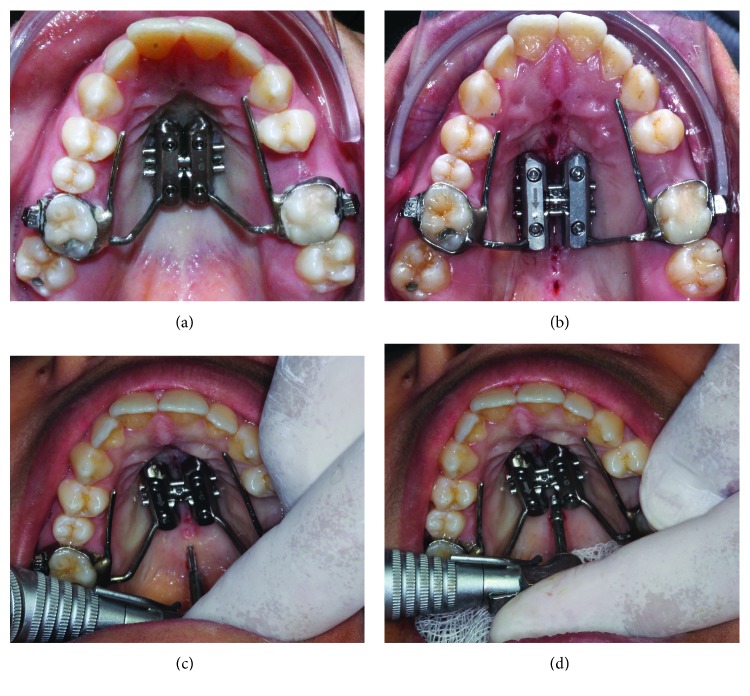
MARPE in place and minimally invasive surgical procedure to reduce suture resistance: (a) day of installation; (b) after corticopuncture procedure (8 perforations); (c) corticopuncture method—first stage: shallow predrilling using lance; and (d) corticopuncture method—second stage: insertion and removal of the miniscrew (4–5 mm depth).

Initial orthodontic planning was to perform rapid palatal expansion with bone anchorage (Figure 4(a)). A MARPE device consisted in an expander screw (9 mm) and 4 self-drilling and self-threading miniscrews. Microimplants were 1.8 mm in diameter, and their length were 11 mm for the anterior ones (7 mm thread and 4 mm neck) and 9 mm in length for the posterior (5 mm thread and 4 mm neck) (Peclab, Belo Horizonte, Brazil). Teeth 16 and 26 were banded and transferred to the impression for laboratory procedures, according to Brunetto et al. [29]. The device was installed using local infiltrative anesthesia and following the procedure, a prescription of antibiotics and analgesic medication; in addition, the use of a chlorhexidine 0.12%-based mouthrinse on for a period of 7 days was recommended.

Activation protocol was 1/4 of activation twice a day for 10 days [29]. However, immediately after the orientation of the activations, the patient reported being unable to perform turn the jackscrew expander due to great resistance to move the key in backward direction. The difficulty was related to the strong resistance of the midpalatal suture, requiring high force to manipulate the expander key from front to back. For this reason, activation attempts were then performed every other day as patient visited the clinic with no success in opening the suture.

#### 2.2.2. Corticopuncture Method

**Figure 5:**
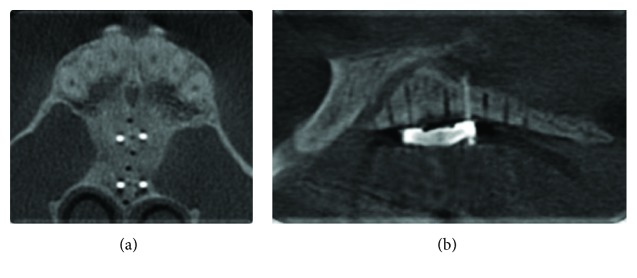
Cone beam CT image of the corticopunctures for an illustration purpose: (a) sagittal view and (b) axial view.

After failing to activate the MARPE device, a minimally invasive surgical method was suggested to the patient in order to reduce suture resistance and accelerate bone remodeling (Figures 4(b)–4(d)). Eight bone perforations, called corticopunctures, were performed along the midpalatal suture. Prior insertion of the miniscrews, cortical bone was shallow manually pre-drilled using a 1.1 mm diameter and 4 mm bur and a contra-angle screwdriver (Peclab, Belo Horizonte, Brazil). Corticopuncture was then performed manually by inserting and removing a 9 mm titanium alloy miniscrew (5 mm double thread, 4 mm neck of length and 1.8 mm diameter). The distance between perforations was 2 mm. It is important to note that corticopunctures were only 5 mm depth in the midpalatal suture. In the anterior part of the palate perforations were done carefully in order to avoid the nasopalatine canal (Figure 5). Corticopuncture procedure was done using greater palatine nerve block anesthesia. After procedure only prescription of analgesic medication for pain relief, in addition to the use of a mouthrinse based on chlorhexidine 0.12% for a period of 7 days.

**Figure 6:**
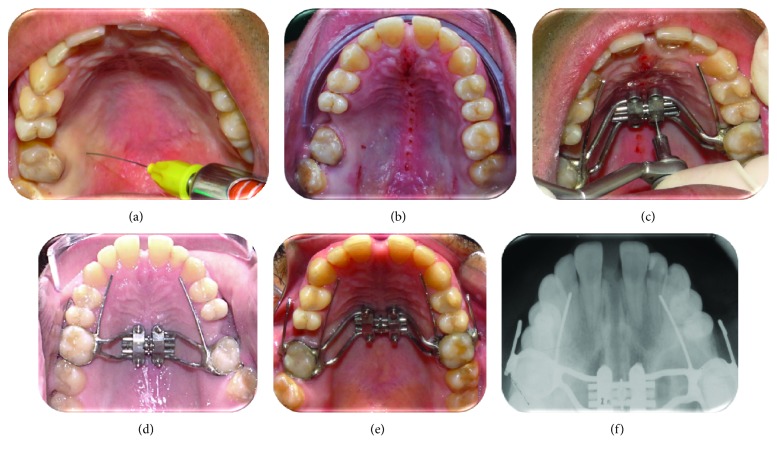
Protocol of the corticopuncture procedure suggested prior MARPE insertion in a second patient: (a) nerve block anesthesia, (b) corticopuncture procedure performed using contra-angle electric screwdriver, (c) maxillary skeletal expander in place and miniscrew insertion, (d) end of procedure, (e) result after expansion, and (f) occlusal X-ray showing midpalatal suture split.

In this patient, corticopunctures could not be performed beneath the jackscrew of the expander since the MARPE appliance was in place, thus limiting the number of perforations. An alternative would be perforating midpalatal suture prior to MARPE and miniscrew insertion.  Figure 6 shows a step-by-step corticopuncture method in a second patient who was treated using 11 mm Maxillary Skeletal Expander I (MSE) (Biomaterials Korea, Seoul, South Korea) and 4 miniscrews (11 mm in length and 1.5 in diameter) [5, 30]. For this patient, a contra-angle electric screwdriver (Model ISD900, NSK, Japan) set in 25 min^−1^ speed and 40 Ncm torque was used to perform the corticopunctures.

#### 2.2.3. Results

**Figure 7:**
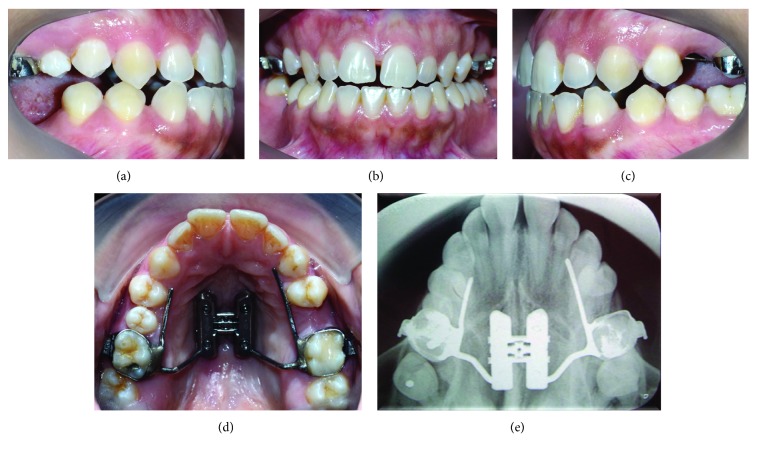
Images of the result after the corticopuncture procedure and second activation protocol: (a) right, (b) frontal, (c) left, (d) upper occlusal view after opening of the medial palatine suture, and (e) upper occlusal X-ray.

After the surgical procedure, the patient was instructed to perform second activation protocol, similar to the one done previously, and better results were obtained, indicating success in splitting midpalatal suture. Images were recorded in the centric relation position, and an occlusal X-ray from the maxilla was taken. A diastema between incisors was then observed (Figure 7).

Cone-beam computed tomography (CBCT) scan using an extended protocol (13 cm FOV and 0.3 mm voxel) was used as the maxilla being the region of interest. Images were generated using OnDemand3D software (Cybermed Inc., Seoul, South Korea) to confirm midpalatal suture split and to evaluate whether the suture opened in a “V” shape or parallel. Later, CT exam will be used to reevaluate and plan the orthodontic phase of the treatment and future evaluation and interdisciplinary planning for implants in the region of teeth 25 and 46.

**Figure 8:**
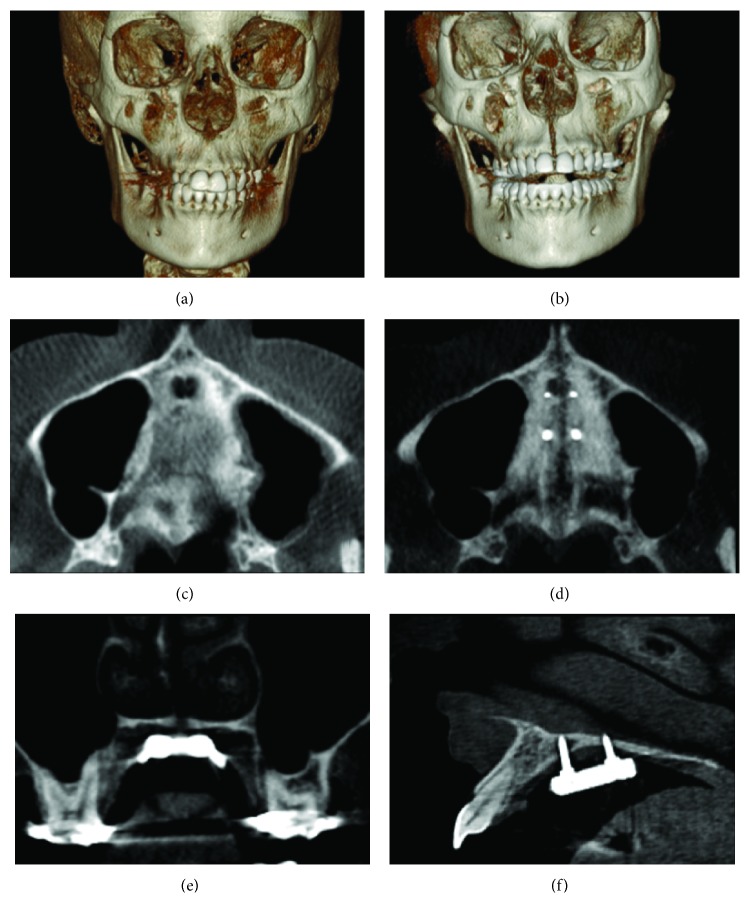
CBCT images after MARPE and corticopuncture procedure: (a, b) 3D reconstructed image in frontal view before and after expansion; (c, d) axial section of the maxilla at the level of anterior and posterior nasal spine before and after expansion; (e) coronal section at the level of the upper first molars showing suture split and molar inclination; and (f) sagittal section showing the bicortical anchorage of the miniscrews.

CBCT evaluation showed parallel split of the midpalatal suture in a coronal view, which means that the amount of opening in the lower portion, near the cervical of the incisors, and in the upper portion of the maxilla, near the nasal cavity, was similar, measuring 2.06 mm of suture separation. In axial view, suture opening occurred more in the anterior region than in the posterior region, connoting a “V” shape. Although molars served as a support for MARPE installation, they did not show evident buccal inclination after expansion, indicating a more skeletal than dental effect (Table 1). An image in a sagittal view close to the microimplants showed that both were anchored in a bicortical engagement of the palate and the nasal cavity floor as recommended by Lee et al. [31] (Figure 8).

**Figure 9:**
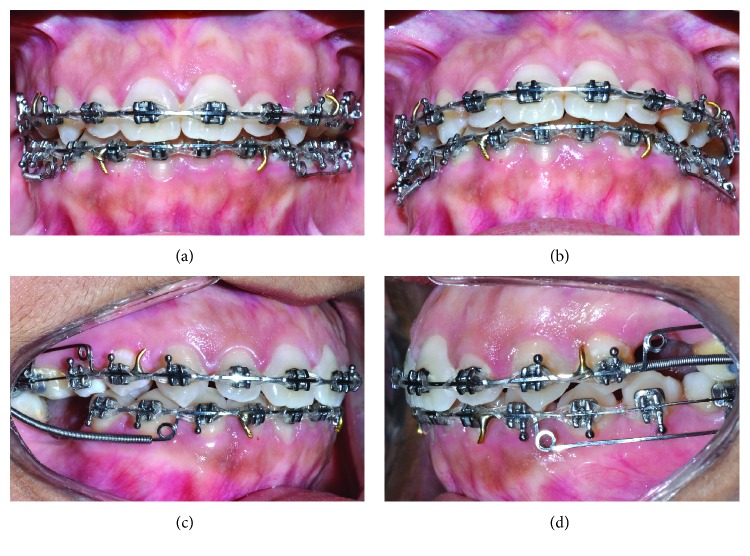
Orthodontic phase of the treatment: (a) frontal, (b) overjet, (c) right, and (d) left.

The orthodontic phase of treatment included the correction of the lower midline deviation, lower right second molar uprighting, intrusion of the upper right first molar, space management for future prosthetic rehabilitation in the missing area of teeth 25 and 46, shape restoration on tooth 15, and torque control (Figure 9).

**Figure 10:**
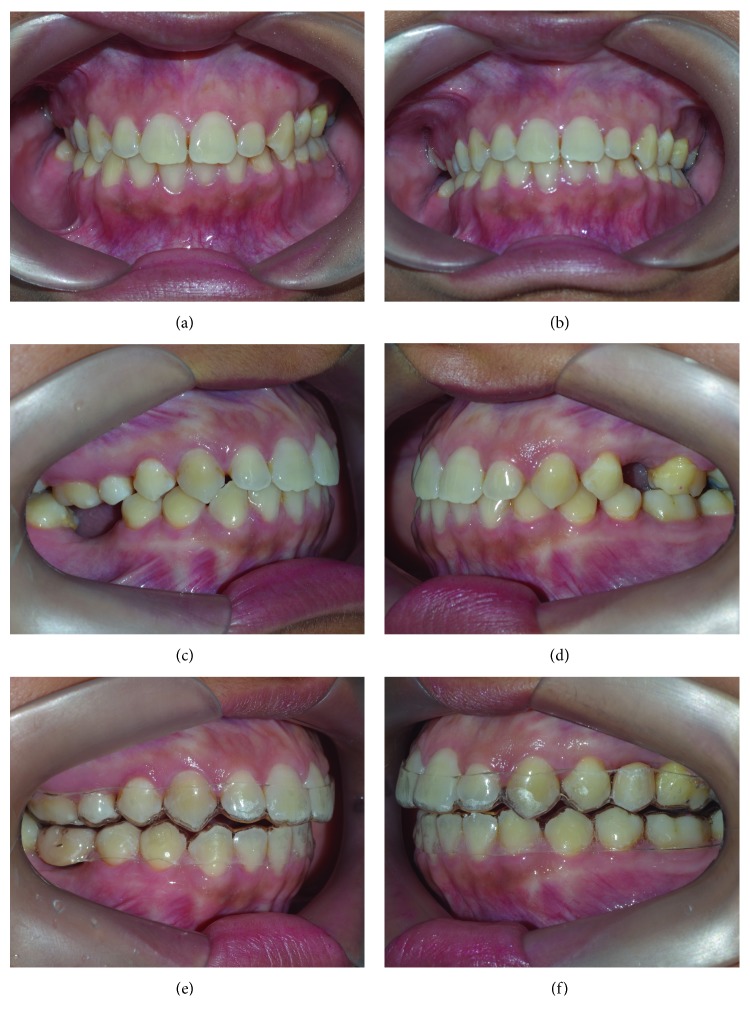
Final pictures: (a) frontal, (b) overjet, (c) right, (d) left, and (e, f) retention appliance with temporary crowns.

**Figure 11:**
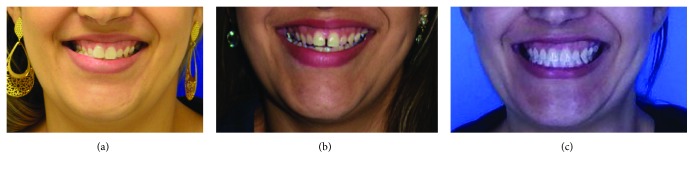
Facial images during smile before, immediately after suture split (presence of the anterior diastema), and after treatment.

Final pictures showed a Class I relationship, good overjet and overbite, and no CR-CO discrepancy. Since spaces were prepared for rehabilitation, the patient was referred to implantology and prosthodontics (Figure 10). Facial pictures show the maintenance of the good profile and improvement of the mandibular deviation in the frontal view.  Figure 11 shows broader smile compared to the initial. Facial photographs during smiling showed before and after suture split, the presence of a diastema between the central incisors immediately after corticopuncture procedure and MARPE reactivations. Final pictures shows an improvement in buccal corridor during smile and also mandibular deviation (Figure 11).

## 3. Discussion

Microimplant-assisted rapid palatal expansion (MARPE) has shown significant results, as seen in the literature review [5, 33–35]. The MARPE device is indicated for the correction of transverse maxillary discrepancy and posterior crossbite, especially in nongrowing patients as an alternative to surgically assisted rapid palatal expansion (SARPE), since rapid palatal expansion may not be an option due to heavy interdigitation of the suture, making it harder to split the two halves of the maxilla conventionally by using tooth-anchored expanders [36]. Although SARPE presents low morbidity, many complications have been reported including hemorrhage, gingival recession, injury to maxillary nerves, infection, pain, devitalization of teeth, sinus infection, and impingement on the palatal soft tissue, among others [16].

MARPE benefits and advantages reported in the literature are described as follows: minimal dental inclination [1, 37, 38]; less risk of damaging effects to the periodontium such as gingival retraction and root resorption [2, 37]; greater vertical control in dolichofacial patients [1]; reduction of tooth movement of anchor teeth [5]; increased protraction of the maxilla when associated to facemask therapy [39]; indicated to patients at the end of the growth and adults [6, 10]; avoidance of orthognathic surgery[5]; stability of the expansion and decrease of relapse [2, 6]; forces applied directly to the midpalatal suture and less amount of force applied to the teeth [40]; and airway improvement [5]. The results of this case report showed that it is possible to correct maxillary constriction in an adult patient using a MARPE appliance which promoted more skeletal effects such as midpalatal suture split and an increase in the basal bone and cortical bone widths than dental tipping, avoiding SARPE procedure in a period of 20 days of activations. The amount of expansion performed for this patient was enough to correct the transverse discrepancy and large buccal corridor, but more expansion could be done using the MARPE device if needed.

In contrast, Garib et al. reported that the MARPE method presented the disadvantage of requiring a longer activation time and twice the force for the rupture of the medial palatine suture compared to SARPE [37]. Other disadvantages are related to the following: it may cause temporary inflammation of the palatal mucosa [2], difficulty in hygiene around microimplants, and risk of infection [1]. Regarding limitations, Choi et al. cited the possibility of failure to separate the suture due to the resistance of the craniofacial structures [6] and when patient presents extremely narrow and deep palate; since proper position of the some of MARPE appliance, such as the MARPE design used in this case report, cannot be achieved, since it should be placed close to the palatal mucosa [29]. Other designs of MARPE expanders may be recommended in the cases of extreme narrow palate but it also may show different clinical results.

Despite the high success rate of MARPE technique, it may be difficult to split midpalatal suture despite bone anchorage, especially in older adults, since the suture may be closely interdigitated, representing a limiting factor for the expansion [12, 14, 41]. This article suggested the use of a surgical method for accelerating bone remodeling to complement MARPE technique in order to facilitate suture split. Pulver et al., in their study on maxillary expansion in rabbits, suggested that greater skeletal expansions may be possible if combined with surgical methods, such as corticotomy, to promote regional acceleratory phenomenon (RAP), stimulating bone remodeling and reducing bone volume and density [42].

Hassan et al. reported that assisted expansion with corticomy, defined as decortication on the buccal and palatal walls of the alveolar bone, has been shown to be an effective technique in the treatment of transverse maxillary deficiency in adults and have suggested that the technique may provide greater stability and better periodontal health than conventional expansion. However, the same study reported that there may be side effects of the corticotomy method such as mild bone loss and loss of inserted gingiva [28]. To avoid this, studies recommend the use of bone grafts to conserve the periodontium [43, 44]. In addition, subcutaneous hematomas and postoperative swelling and discomfort were also associated with the corticotomy procedure [28].

In order to minimize the surgical procedure and reduce postoperative discomfort, other techniques may be recommended. Micro-osteoperforation is a method that increases the expression of cytokines and chemokines responsible for stimulating the differentiation of osteoclasts in bone remodeling and thus increasing the rate of tooth movement by up to 62% [21]. Piezocision showed a greater number of osteoclasts along the surface of the alveolar bone, with consequent acceleration of tooth movement [23]. Similar results were found in corticision [19] and, more recently, using corticopuncture [24]. Tsai et al. (2016) compared the effects of corticotomy and bone microperforations and concluded that both techniques increased bone remodeling and there were no significant differences between them.

This case report evidenced the possibility of failure of the MARPE technique in splitting the midpalatal suture, and to the best of our knowledge, this is the first case report demonstrating the benefit of applying a minimally invasive surgical method to accelerate bone remodeling, such as corticopunctures. Corticopunctures performed along the midpalatal suture allowed its opening which has not been achieved by the conventional protocol for MARPE activation. Although the method resulted in the increase in the dental archwire perimeter, correcting crossbite with minimal side effects, after 10 days of activation, resistance to activate the jackscrew was once again observed. This observation may be related to the expander design used in this case report which required a stronger and stiffer activation key, stronger key system such as MSE II design (Biomaterials Korea, Seoul, South Korea), or even new corticopunctures to be performed. Also, it is known that other areas of resistance can play a role during maxillary expansion such as piriform aperture pillars (at the anterior region), zygomatic buttresses (laterally), and pterygoid junctions (posteriorly) [15]. More studies are needed in order to determine the optimal clinical protocols and the skeletal effects of this corticopuncture method associated with the rapid expansion of the maxilla assisted by microimplants.

## 4. Conclusion

A minimally invasive surgical procedure named the corticopuncture method as an adjunct to the MARPE technique may be beneficial in adult patients who may present resistance of the midpalatal suture and adjacent sutures due to the high interdigitation of these structures.

## Figures and Tables

**Table 1:** CBCT measurements before and after expansion [32].

Measurements	Initial		After expansion	
	Premolar	Molar	Premolar	Molar
Basal bone width (mm)	42.54	59.35	46.84	62.38
Cortical bone width (mm)	46.45	57.41	50.88	60.51
Midpalatal suture split (mm)	0	0	3.14	2.06
Tooth inclination (degree)	85.2° right 90.6° left	89.4° right 92.1° left	86.5° right 91.5° left	90.3° right 93.8° left
